# Hip Labral Reconstruction With a Synthetic Graft: Development and Preclinical Validation

**DOI:** 10.1111/os.70246

**Published:** 2026-03-20

**Authors:** Enrico Tassinari, Mauro Petretta, Giorgia Borciani, Luca Cristofolini, Eleonora Olivotto

**Affiliations:** ^1^ 2nd Orthopaedic and Traumatologic Clinic, IRCCS Istituto Ortopedico Rizzoli Bologna Italy; ^2^ RegenHu Company Villaz‐Saint‐Pierre Switzerland; ^3^ RAMSES Laboratory, RIT Department, Research Centre Codivilla‐Putti IRCCS Istituto Ortopedico Rizzoli Bologna Italy; ^4^ Department of Industrial Engineering Alma Mater Studiorum‐University of Bologna Bologna Italy

**Keywords:** biomechanical testing, femoroacetabular impingement, hip arthroscopy, osteoarthritis, synthetic graft for the acetabular labrum

## Abstract

**Objectives:**

Hip Osteoarthritis (OA) affects a significant component of the adult population, placing itself among the most important causes of disability and need for total hip replacement. Femoroacetabular impingement (FAI) is an anatomic alteration of the proximal femur and/or acetabulum leading to chondro‐labral damage and playing a prominent role in OA pathogenesis. Thus, treating FAI is fundamental to relieve hip pain and further joint tissue deterioration. Labral reconstruction is considered the treatment of choice, in particular using tendon allografts or autografts which, however, have some limitations. Here, we investigated the possibility to create a synthetic graft for labral reconstruction to best restore the load bearing and ready to be used in the surgical room.

**Methods:**

The graft was designed analyzing the anatomical structures from intact tissue samples. After a preliminary screening of different polymers, silicone was selected for its flexibility and elasticity to better adhere to the implantation site. We used an FDA‐approved biocompatible silicone (VK100), and a mold casting was selected as a fabrication method. Cytocompatibility of VK100 was tested in vitro with an immortalized chondrocytes human cell line (C‐28/I2). A cadaver lab was used to test the implantation procedure and to investigate the effects of the device transplantation on hip range of motion, translation, and resultant joint stability. To test the long‐term strength of the reconstruction under cyclic loading, synthetic hemipelves were prepared for biomechanical testing and subjected to 10,000 cycles where deflections of up to 5 mm were imposed.

**Results:**

In vitro tests showed that up to 14 days of culture C‐28/I2 cells were alive and adherent to VK100 surface with the formation of cell protrusions. As for cell cytotoxicity, a slight increase in LDH levels was observed at 14 days, probably due to the high confluence of adherent cells. We also demonstrated with the ex vivo procedure on cadaver that the device was suitable for arthroscopic implantation without damage or structural compromise during fixation to the acetabular bone. The range of motion and joint stability were preserved after implantation. Furthermore, the graft reconstruction successfully passed strenuous biomechanical cyclic loading. The force peak decreased by less than 10% during the test, indicating no detectable reduction of stiffness nor displacement/failure of the graft. No sign of damage was observed after test completion.

**Conclusions:**

Overall, these results suggest that we have developed a functional synthetic graft that might be quickly transferred to clinical practice.

## Graphical abstract

Femoroacetabular impingement plays a prominent role in osteoarthritis pathogenesis. Treating FAI is fundamental, and labral reconstruction is considered the treatment of choice. A synthetic graft ready to be used in the surgical room for labral reconstruction, to best restore hip function, might be the solution.

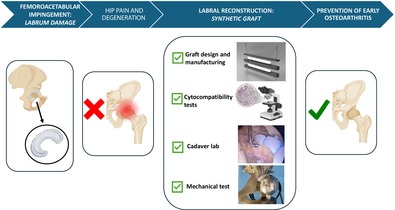

## 1 Introduction

Femoroacetabular impingement (FAI) is a pathological mechanical process, caused by anatomic abnormalities of the proximal femur and/or acetabulum that result in repetitive collisions occurring during dynamic hip motion that lead to regional loading of the femoral head–neck junction against the acetabular rim [1, 2, 3]. In the athletic population, as the functional motion requirements in many sports exceed the physiological one, the anatomic deformities induce conflicts particularly relevant [4, 5]. The resulting abnormal kinematics can lead to direct injury to the cartilage, labrum, and surrounding capsular structures, causing pain and loss of function, and ultimately resulting in early osteoarthritis (OA) [6].

Hip arthroscopy has become an important surgical intervention for the treatment of many patients with early hip disease and has grown in popularity at an exponential rate over the past years [7, 8]. In particular, FAI represents the most common indication for arthroscopic intervention [9, 10, 11] with the focus of preserving the acetabular labrum as it is critical to maintaining not only joint stability but function itself [12].

Since the turn of the century, there has been an evolution in the treatment of the acetabular labrum during hip arthroscopy. While initial studies pointed to labrum debridement as the first option in labrum lesions treatment [13], the current consensus supports labrum repair/refixation to preserve its anatomy [14, 15, 16]. However, recently, there has been a paradigm shift toward labrum augmentation and reconstruction to treat hypoplastic and irreparable labra, respectively [17, 18, 19, 20].

Early results have been promising, as labrum reconstruction is considered the treatment of choice in hip arthroscopy reviews [21], particularly as surgeons seek a solution for patients who are in pain after labrum debridement. Some authors have also advocated its use in primary hip arthroscopy [22, 23] but this remains challenging due to the difficult diagnosis.

To date, labrum reconstruction techniques use tendon allografts or autografts [24, 25], previous studies have demonstrated their efficacy in short‐term outcomes in the primary general and active populations [26, 27]. About the rate of success for mid‐ to long‐term outcomes, a systematic review [28] recently describes only one study with a 5‐year average of follow up (FU), three studies with a minimum of 5 years of FU, and one study with a 10‐year minimum FU. All 5 studies (219 hips in total) demonstrated significant improvements reported in Patient‐Reported Outcomes (PROs) (in particular the modified Harris Hip Score), with a conversion range to total hip arthroplasty from 0% to 36%.

Nevertheless, both allografts and autografts have limitations mainly related to various factors including: the shape of the tendon, which is circular in cross‐section and cannot grant an efficient sealing effect of the joint, resulting in altered function [29]; the handling and preparation of the graft (both autologous and homologous) which lead to further lengthening of the surgical time [30]; the need for an additional skin incision (i.e., autograft) to harvest the *fascia lata* or hamstrings or others [31].

As for the use of allograft versus autograft, a recent systematic review of comparative studies [31] shows no differences in most reported outcome measures. Both techniques have similar limitations: they don't recreate the original labrum's shape; harvesting (in case of autograft), handling, and preparation of the graft lead to further lengthening of the surgical time and a not irrelevant risk of infection in the sampling site.

About the use of synthetic graft for hip labral reconstruction, only the use of a polyurethane (PU) scaffold (normally used for meniscus transplantation) is mentioned in literature [32]. At 4 years after implantation, Tey‐Pons et al. [32], showed in three patients an improvement in hip joint function, reduced pain, and scaffold preservation on follow‐up imaging. The main limitation of the present study was the small sample size. Moreover, the augmentation was limited to the frayed or manipulated labrum for pincer resection but not extended to the complete hypoplastic labrum.

There is therefore a need to find a biomaterial, flexible and elastic for better adhesion to the implantation site, that covers the reconstruction of most lesions. A synthetic graft represents more than an incremental improvement over current reconstructive techniques introducing the possibility to overcome all those limitations with a customizable, off‐the‐shelf biomimetic implant with defined mechanical properties.

The aim of the present study is to develop a synthetic graft with an anatomical profile to best restore the load bearing, ready to be used in the surgical room for a more effective and rapid surgical technique. For this reason, the following objectives were tackled: (i) design and modeling of the three‐dimensional (3D) structures and investigation of different materials to identify the most suited formulation to match mechanical and clinical needs; (ii) fabrication of the devices; (iii) in vitro biological characterization of the selected material; (iv) surgical implantation procedure simulation on cadaver; (v) endurance testing of the graft reconstruction to strenuous cyclic biomechanical loading.

The final goal is to quickly transfer the synthetic graft to clinical practice since it might be applicable for reconstruction of the largest number of lesions (both segmental and circumferential).

## 2 Methods

### 2.1 Graft Prototype Design

The graft design was carried out in conjunction with surgeons, and analyzing the anatomical structures observed from intact tissue samples discarded from FAI patients undergoing hip arthroscopy and multi‐organ donors. The average measures of the acetabular labrum were also compared to the existing literature [33] and to the allograft prepared with fascia lata for the circumferential (or front‐to‐back) technique for labral reconstruction [34]. Moreover, to create a suitable graft easily adaptable by the surgeon to all lesion sizes, the length was fixed to the greatest average length of 100 mm.

**FIGURE 1:**
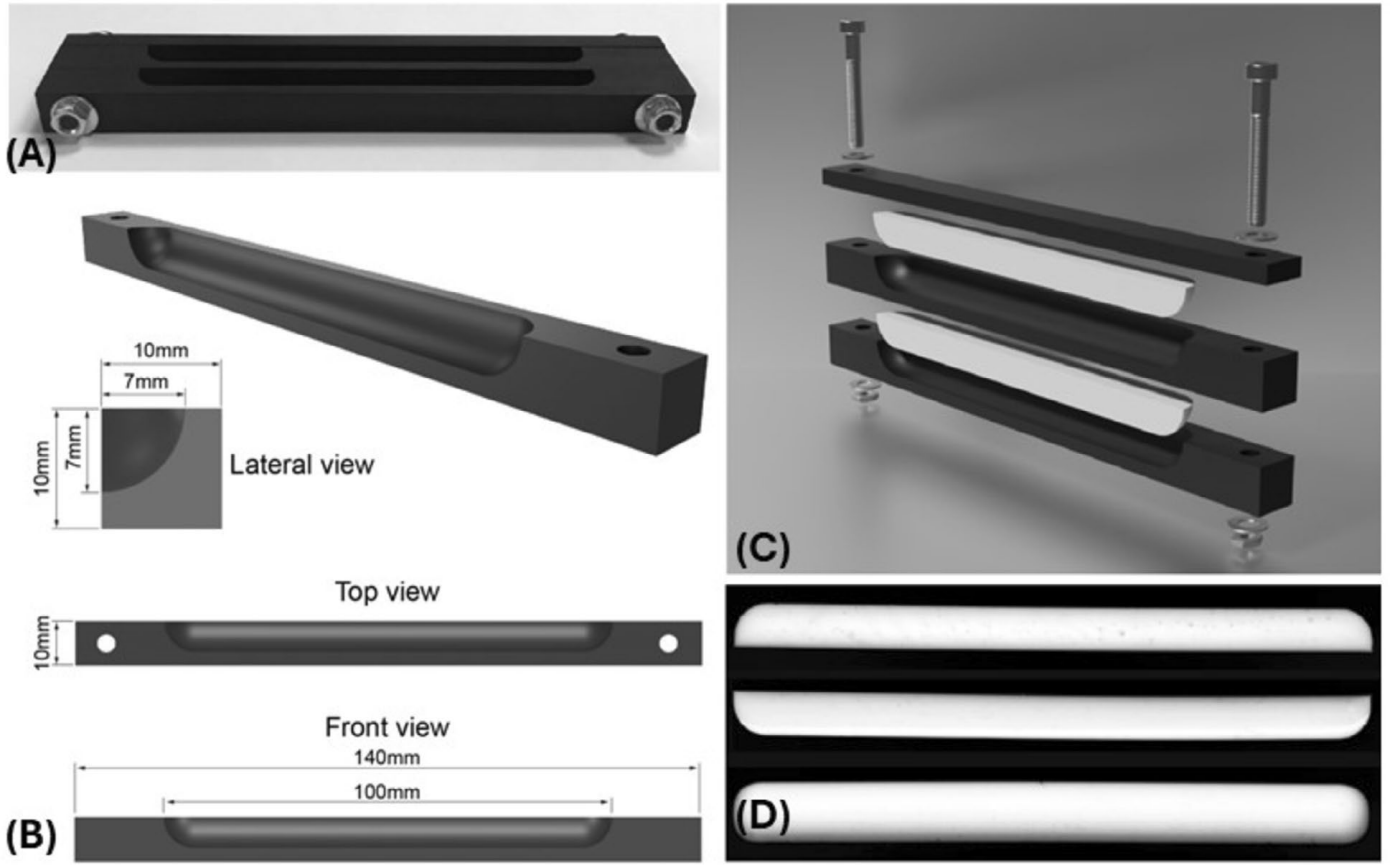
Teflon negative mold of the graft. (A) Photograph of the mold produced by milling. (B) Technical drawings showing the main dimensions in lateral, top, and front views. (C) Rendering of the exploded view of the mold and two silicone grafts. (D) Photograph of the assembled mold (lateral and upper view respectively).

The graft was modeled as an axially extruded block with a cross‐section characterized by two perpendicular flat surfaces joined by a curved segment in the shape of a circular arc (Figure 1), designed to increase adhesion to the implantation site (Autodesk Inventor, San Francisco, CA, USA).

### 2.2 Preliminary Exploration of the Manufacturing Options

A range of polymers was preliminarily explored to identify the best one for this application. Polycaprolactone and Polyurethane, which are commonly used in biomedical applications, were first considered, using a 3D printing strategy. These first prototypes were used for preliminary tests ex vivo on a cadaver pelvis (data not shown here for brevity). However, they were soon discarded as they did not provide the desired deformability and elasticity.

Silicone was eventually selected to match the flexibility and elasticity of the acetabular labrum. Due to their unique properties and advantages over thermoplastic polymer formulations, silicone‐based biomaterials have in fact been considered for meniscal and labral replacement applications [35, 36]. The first proof of concept was produced using commercially available silicone, not suited for medical use.

### 2.3 Silicone Prototype Manufacturing

As the goal of the study is to develop a product that could be readily transferred to clinical application, a second trial was performed selecting an FDA approved biocompatible silicone already used in clinical practice for spine surgery [37, 38], pre‐filled VK100 cartridge (ST.VBX‐0125 lot. 2023082490, BONWRx, Lansing, Michigan, USA).

Given the different rheological properties of the selected biomaterial formulation, to guarantee an optimal shape fidelity for the second generation of prototypes, a mold casting approach was selected as a fabrication method. A Teflon negative mold of the graft was produced by milling as a cast for the prototype (Figure 1A–C). The VK100 silicone was poured slowly into the mold to avoid trapping air bubbles, filling it completely. At the end of the procedure, the upper surface of the samples was leveled with a steel spatula, and the samples were cured for 24 h to reach the final mechanical properties and guarantee shape retention and stability.

### 2.4 Cell Culture, Toxicity, and Viability Assays

The cytocompatibility of the silicone elastomer VK100 was previously evaluated using the Murine MC3T3‐E1 preosteoblast cell line [39], where Song W et al. assessed the potential toxicity of VK100 monomers released in the culture medium absorbed on a soaked filter article placed directly onto a semiconfluent cell monolayer and incubated for 24 h.

In our study, we tested the cytocompatibility of VK100 with the direct contact of the silicone elastomer with the immortalized chondrocytes human cell line C‐28/I2 [40, 41]. The cells were maintained in culture in Dulbecco's Modified Eagle Medium (DMEM)/F12 (1:1), 1% penicillin–streptomycin (11330‐032 and 15140‐122 respectively, Gibco—Thermo Fisher Scientific, MA, USA) and 10% fetal bovine serum (F7524, Sigma‐Aldrich, MO, USA) (hereafter referred to as DMEM complete).

VK100 samples, with 5 × 5 × 1 (Length, Width, Height) mm dimensions, were sterilized in autoclave and pre‐wetted by immersion in DMEM complete for 3 h (*n* = 3). Then, a suspension of 200,000 cells/mL was prepared, and 1 mL was pipetted carefully on VK100 samples to allow cells to remain on the upper surface. Cells were allowed to adhere for 2 h in incubator, then DMEM complete was gently added to cover the cell‐loaded samples. Samples were maintained in culture up to 14 days (d), at 37°C, 95% humidity, with medium changes every 3 days.

Cell morphology and adhesion were observed by fixing cells with 3.7% paraformaldehyde for 2 h and performing hematoxylin and eosin (H&E) (Bioptica, Milano, Italy) staining. Live&Dead assay was also performed to test cell viability (LIVE/DEAD Viability/Cyto‐209 toxicity Kit, Thermo Fisher Scientific) according to manufacturer instructions. The test is based on the simultaneous determination of live (green) and dead (red) cells with two specific probes, calcein AM and ethidium homodimer (EthD‐1), respectively [42].

All images were captured using a Nikon Eclipse 90i microscope equipped with Nikon Imaging Software elements (Nikon Corporation, Minato, Tokyo, JP).

To evaluate cell metabolism and quantify potential cytotoxicity, Lactate Dehydrogenase Colorimetric Activity Kit (LDH) (Roche, 11644793001) was performed following the manufacturer's instructions. To better monitor the potential cytotoxic activity of VK100 on cells at the first days of culture, a timepoint of 3 days of culture was added. The samples' absorbance was quantified by using a microplate spectrophotometer (Infinite F200 PRO, TECAN, Mannedorf, Switzerland) at 490 nm wavelengths. Measurements were performed in triplicate.

### 2.5 Cadaver Lab and Surgical Technique

The first fabricated devices were tested ex vivo on a cadaver pelvis by an experienced hip surgeon to obtain some preliminary feedback from the surgical team concerning the implantation procedure. The focus of the cadaver lab was to test the feasibility of the surgical procedure and to assess the immediate post‐operative effects of the implantation of the device in terms of hip range of motion, translation, and resultant joint stability. In particular, similar to what is done intra‐operatively during other hip procedures, the surgeon imposed flexion‐extension, abduction‐adduction, and internal‐external rotation to assess that the range of motion was not constrained. Similarly, traction forces were applied in all directions to qualitatively assess the stability of the operated joint.

**FIGURE 2:**
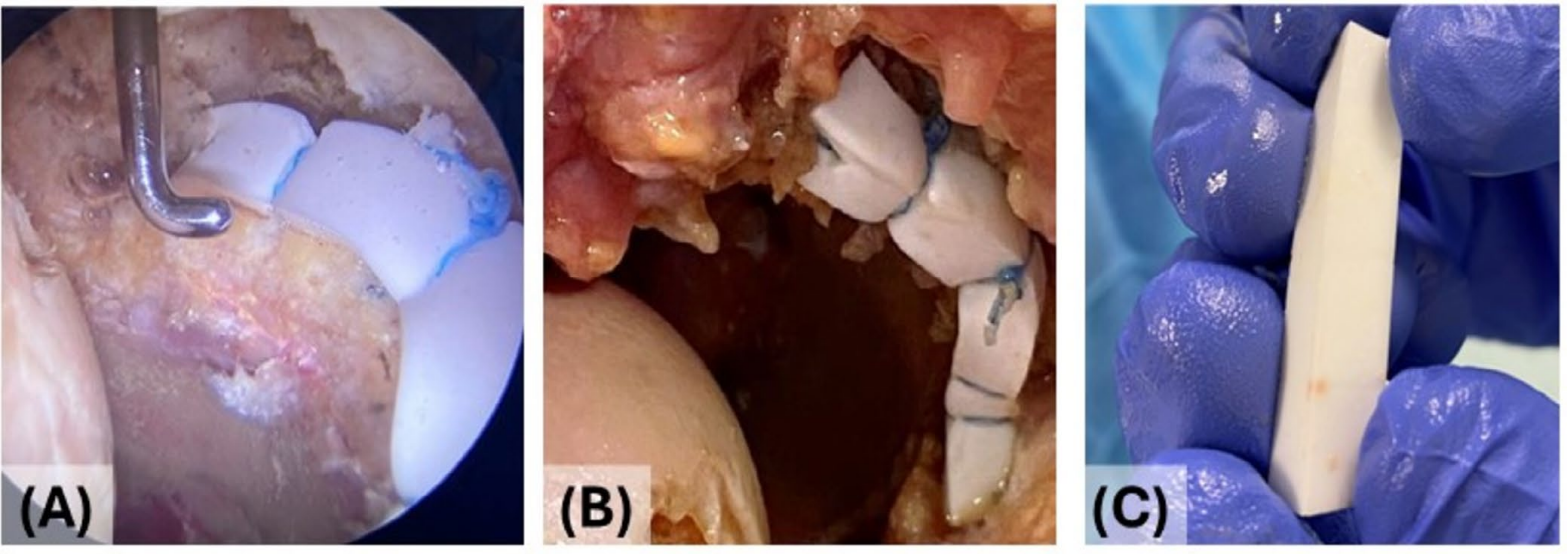
Cadaver lab and surgical technique. (A) Arthroscopic image of the graft implantation using four suture anchors (Q‐FIX, 1.8 mm all‐suture anchor, Smith & Nephew Endoscopy, Andover, MA) in a pelvis acetabulum from cadaver. (B) Image of the implanted graft after dissection of the anatomical hip specimen. (C) Image of the graft removed from the hip after the cadaver lab showing no macroscopic damage or visible structural issues.

With the hip under traction by two arthroscopic portals (Antero‐Lateral and Mid‐Anterior), the device was positioned from 10 o'clock to 1 o'clock (left hip) using four suture anchors (Q‐FIX, 1.8 mm all‐suture anchor, Smith & Nephew Endoscopy, Andover, MA) following “Kite” technique [43, 44] (Figure 2A,B). All the procedures were performed at the ICLO Teaching and Research Center (ICLO St. Francis de Sales, Verona, Italy).

### 2.6 Mechanical Test

Similar to what is prescribed for other hip devices (e.g., by the ISO 7206 standards), a pass/fail test was designed. In order to test the strength of the reconstruction under cyclic loading, hemipelves were prepared for biomechanical testing (*n* = 4). The simplified test conditions were designed to maximize reproducibility while granting biomechanical relevance. As cadaver bone would easily get damaged due to cyclic loading, synthetic models were chosen (Mod. 3405, 4th Generation composite, Pacific research Labs, Vashon Island, WA, USA). They consist of a shell of glass‐fiber reinforced epoxy to replicate the cortical bone and a polyurethane foam core replicating the trabecular bone. These models have been validated and extensively used to replicate the mechanical behavior of the human pelvis [45]. Each specimen was aligned in a reliable reference frame [46] and potted in an aluminum pot with bone cement. The surgeon prepared four identical specimens using the same surgical technique previously optimized and used in the cadaver lab reported in the previous paragraph [43, 44].

The labrum itself is not a load‐bearing structure. Indeed, while forces of the order of several body‐weights are transferred by the hip through the articular cartilage, the labrum has to deform to second the deflection imposed by the femoral neck and head when the end of the range of motion is reached [1, 5, 47, 48].

**FIGURE 3:**
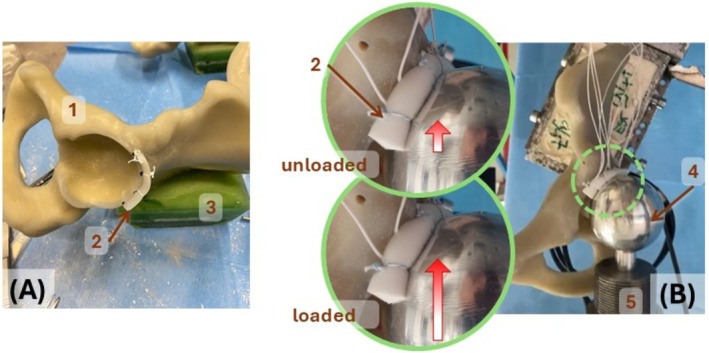
Mechanical test. (A) Image of a synthetic hemipelvis (1) with the graft implanted (2), ready for the biomechanical test (*n* = 4). The same surgical technique used in the cadaver lab was replicated. Also visible is the cement pot (3) used to constrain the specimen during the mechanical tests. (B) Overview of the testing setup where a spherical metal head (4, representing the femoral head), mounted on the actuator (5) of the testing machine, was cyclically pressed against the labrum. Each specimen was subjected to 10,000 cycles, with an imposed displacement of between 2.0 and 5.0 mm. The rounded inserts show the force direction and highlight the graft deformation in the unloaded (top) and loaded (bottom) conditions.

The hemipelves were tested in a universal testing machine (mod. 8032 with 8800 controller and 10 kN load cell, Instron, Canton, MA, USA). The worst‐case scenario was simulated, where the femoral head–neck impinged against the reconstructed labrum (Figure 3). The hemipelves were fully constrained through their cement pot, while a metal sphere having the same diameter as the ideal femoral head was brought in contact against the reconstructed labrum, in the cranial direction. The loading protocol was designed to simulate a very demanding condition, representative of an extremely severe in vivo use (i.e., more demanding than observed in FAI patients [47]). For this reason, the “head” was offset by 20 mm with respect to the anatomical position. For each specimen, the following test sequence was applied:

– The metal head was brought in contact with the labrum (preload = 20 N).
– Ten conditioning cycles (haversine of 2.0 mm at 1 Hz) were applied, then the preload (20 N) was restored.
– To simulate the case where the femoral head neck impinges against the reconstructed labrum, 10,000 cycles of constant displacement amplitude were imposed while the force was monitored.
– To test increasingly demanding conditions, the four specimens were respectively tested with 2.0, 3.0, 4.0, and 5.0 mm displacement. The magnitude of such displacement was kept constant throughout the 10,000 cycles for each of the specimens. The magnitude of these displacements corresponds to those classified as “low” up to “high” in [49], and are consistent with those reported in [48].
– The force at the beginning of the test and throughout the test was monitored to detect possible failures due to the cycling loading.
– The force at the end of the test and after 1 h was measured to assess if any permanent deformation of the reconstructed labrum had occurred.

Therefore, the output of the test was mainly a pass/fail (based on integrity or failure) with some additional information about force variations.

### 2.7 Statistical Analysis

Tukey's multiple comparisons test was used to analyze the LDH data using GraphPad Prism 10 v. 10.0.2 (GraphPad Software LLC, Boston, MA, USA).

## 3 Results

### 3.1 Silicone‐Based Graft Prototypes Production

Prototypes of the acetabular labrum graft made of VK100 silicone were successfully fabricated by mold casting approach as shown in Figure 1D.

### 3.2 Cytocompatibility of VK100 on Human Chondrocytes Cell Line C28/I2

**FIGURE 4:**
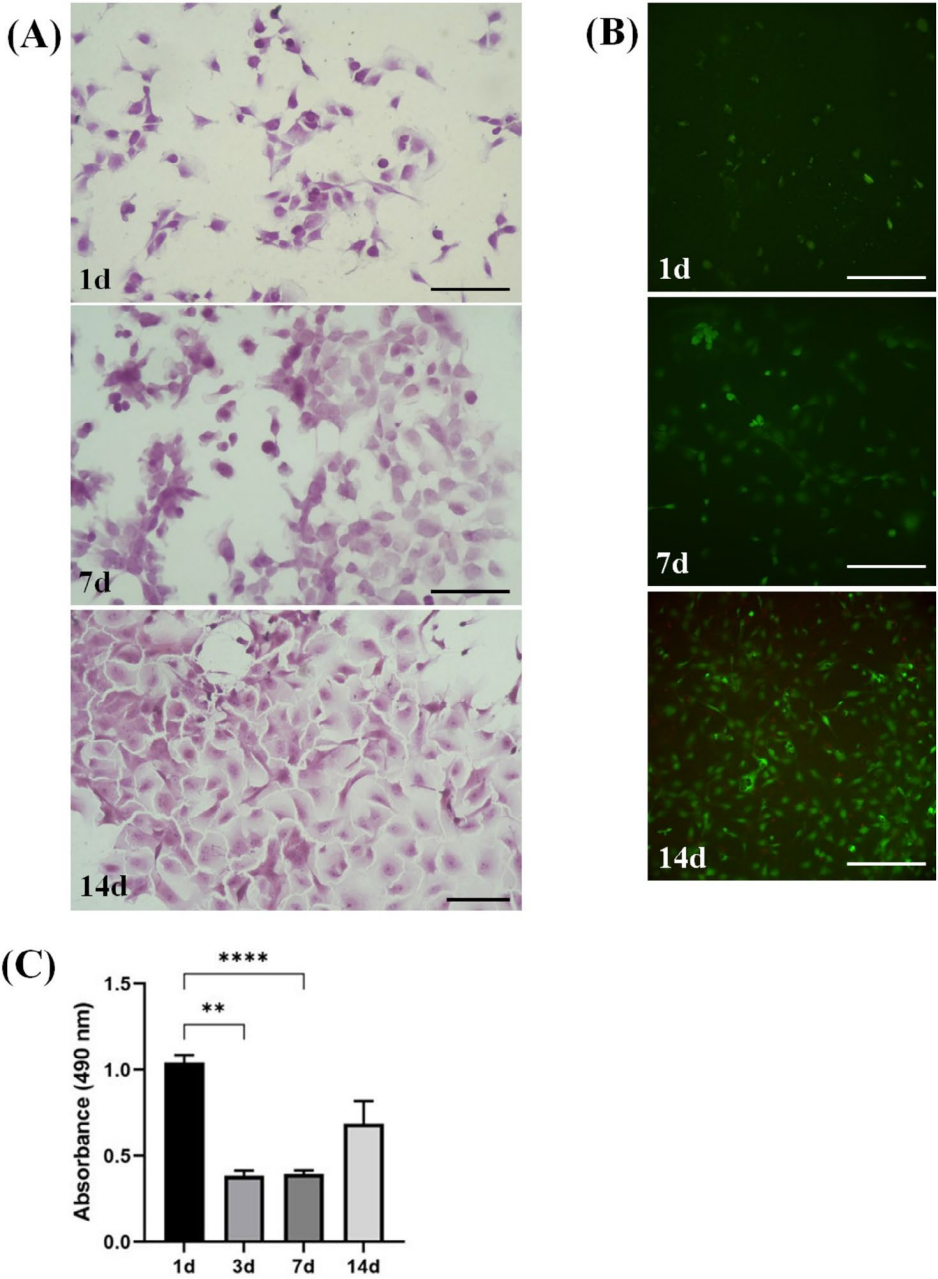
Cytocompatibility tests of VK100 on immortalized chondrocytes human cell line C‐28/I2. A suspension of 200.000 cells/mL was seeded on VK100 samples 5 × 5 × 1 (Length, Width, Height) mm dimensions. Samples (*n* = 3) were maintained in culture up to 14 days (d), at 37°C, 95% humidity. (A) Images captured at light microscope at 1–7–14 days of culture showing C‐28/I2 cells morphology stained with Hematoxylin&Eosin and adherent to VK100 surface with the formation of cell protrusions and filaments (bar = 100 μM). (B) Live&Dead assay was performed to test cell viability at 1–7–14 days of culture: The test is based on the simultaneous determination of live (green) and dead (red) cells with two specific probes, calcein AM and ethidium homodimer (EthD‐1), respectively. For each timepoint most of the cells were alive (bar = 100 μM). (C) Quantification of Lactate Dehydrogenase (LDH) release in cell medium tests the potential toxicity of VK100. LDH release after 1 day from the seeding, probably due to cellular stress. After 3 and 7 days of culture, only a negligible level of LDH release was detected. After 14 days, a slight increase in LDH levels was observed, probably due to the high confluence of adherent cells (**p = 0.0028; ****p < 0.0001).

After 1 day of culture, C‐28/I2 cells were adherent to VK100 surface with the formation of cell protrusions and filaments (Figure 4A). At later timepoints, an increased number of cells adherent to the biomaterial surface can be appreciated, up to 14 days where most of the available surface was colonized by cells. The Live&Dead test confirmed that for each timepoint most of the cells were alive (Figure 4B).

Concerning cell cytotoxicity, C‐28/I2 cells showed the higher release of LDH at the first timepoint, that is after 1 day from the seeding, probably as a result of cellular stress. After 3 days and 7 days of culture, only negligible levels of LDH release were detected, while at 14 days, a slight increase in LDH levels was observed, probably due to the high confluence of adherent cells (Figure 4C).

### 3.3 Silicone Graft Application in Arthroscopic Procedure

During the arthroscopic procedure in a pelvis from cadaver, the device showed significant ductility and suitability for arthroscopic implantation, without damage or structural compromise during fixation to the acetabular bone via high‐strength suture threads. At the end of the procedure, the dissection of the anatomical hip specimen was performed, revealing secure and correct anchorage of the device to the acetabular bone (Figure 2B), with no macroscopic damage or visible structural issues (Figure 2C). The surgeon performed the operated hip FADIR [50] and FABER test [51]. When forces were applied in all directions, the operated joint was stable with no sign of a tendency to dislocate.

### 3.4 Mechanical Endurance of the Graft

All the hemipelves with the silicone labrum reconstructions withstood the cyclic test with no macroscopic failure. The force peak measured at the end of the cyclic test decreased by less than 10% compared to the force measured at the beginning of the test (within 10%). This confirms that no detectable displacement or failure of the reconstruction and no remarkable reduction of material stiffness has occurred. The small reduction of force peak observed at the end of the test was recovered (within 2%) after 1 h after test completion. This confirms that the little variation observed was due to the natural viscoelastic (recoverable) deformation of the labrum, rather than damage.

After test completion, the specimens were inspected also with the aid of a magnifying lens, focusing on possible indents on the surfaces, scratches on the edges, and damage on the stitches. No sign of damage could be detected, confirming that the test was passed. Representative high‐resolution images are provided in the Supporting Informations.

## 4 Discussion

In this study, we present a synthetic graft as a potential option to overcome the previously discussed limitations. This graft is designed to be ready‐to‐use in the surgical room, thus avoiding the need for a second intervention and enabling a more efficient surgical workflow.

The primary goal of labral lesions surgical treatment is to reduce pain and improve the functions of the labrum, thereby maintaining the overall hip stability. In general, both autografts and allografts grant clinically significant improvements in particular in short‐term outcomes [26, 27] and with a promising rate of success for mid‐ to long‐term outcomes with a conversion range to total hip arthroplasty from 0% to 36% [28], although future research efforts should focus on determining the optimal graft choice [30].

Despite their clinical utility, allografts or autografts [24, 52] have several limitations mainly related to the shape of the tendon scaffold, its handling and preparation which result in increased surgical time [24, 29, 30]. Additional limitations of the actual reconstruction techniques are related to the extended traction times during the surgery, technical difficulties in graft insertion and fixation, and the risk of iatrogenic injury [53].

In this study we present a synthetic graft as a potential option to overcome the previously discussed limitations. This graft is designed to be ready‐to‐use in the surgical room, thus avoiding the need for a second intervention and enabling a more efficient surgical workflow.

The benefits of synthetic graft are multiple both for the patient's wellness and for the public health care costs. First, there is no risk of disease transmission or immune rejection as in allograft transplantation. On the economic side, they require only one procedure instead of the two needed for natural grafts for a more effective and rapid surgical technique.

### 4.1 Graft Prototype Design and Manufacturing

The design process was conducted by a multidisciplinary team of bioengineers, biologists, and orthopedic surgeons, reproducing the anatomical structures observed from intact native tissue samples [54]. The design was tailored to ensure adequate mechanical and biological performance, and special consideration was given to translational aspects such as promoting the interaction with host tissue and facilitating the surgical procedure. An FDA‐approved biocompatible silicone, already used in clinical practice for spine surgery, was selected to develop a product readily transferable to clinical application.

### 4.2 Cytocompatibility of the Silicone Elastomer

Moreover, the in vitro tests showed that C‐28/I2 cells adhered to VK100 surface over a period of 14 days, with most of the available surface colonized by cells. The Live&Dead test also confirmed a notable number of viable cells and a negligible number of dead ones. VK100 did not show any cell cytotoxicity, even at high cell confluence, indicating cytocompatibility under physiological conditions [55].

### 4.3 Prototype Test in Arthroscopic Procedure

The cadaver lab allowed testing feasibility of the surgical procedure. The graft was ductile and resistant during the arthroscopic procedure in the cadaver lab, and it did not show any macroscopic damage or visible structural issues after collection at the end of the procedure, confirming the potential applicability on patients. The cadaver lab also allowed assessing the short‐term performance: the just‐operated cadaveric hip joint was stable, while the range of motion was uncompromised.

### 4.4 Mechanical Endurance of the Graft

Interestingly, the mechanical tests (which replicated a very challenging condition), with strain levels exceeding those usually observed in FAI [49] confirmed no macroscopic failure after the 10,000‐cycles test. The small reduction of stiffness (observed as a variation of the force peaks) was due to the natural viscoelastic (recoverable) deformation of the labrum, rather than damage. Indeed, Ferguson et al. have shown the importance of elastic spring‐back of the labrum to preserve its function [56].

### 4.5 Limitations

It must be noted that the mechanical tests mainly focused on the integrity of the reconstructions after testing whereas limited information was collected about other possible parameters (e.g., load capacity, energy dissipation). While this can be seen as a possible limitation of the method, it did not prevent assessing whether the reconstructions would undergo failure under cyclic loading. Indeed, a similar pass/fail approach is followed for many other hip devices (e.g., as prescribed by the ISO 7206 standard). As the labrum is crucial in retaining a layer of pressurized intra‐articular fluid for joint lubrication and load support/distribution, contributing to hip stability through its suction effect, further studies will be focused on suction‐seal/tribology experiments.

Concerning the cadaver lab test, it was limited to short‐term feasibility as it was focused on the surgical procedure. To evaluate possible events of long‐term implant failure and the compatibility of these approaches with the required clinically approved materials, in vivo studies and clinical trials in human subjects are required to fully assess the graft's performance over time. Further improvements could be envisioned in the future related to the prototype architecture, through continuing interaction with the surgical team, to optimize the implantation procedure outcome and minimize its execution time and related risk.

### 4.6 Prospects of Clinical Application

Another important aspect to be considered for clinical translation is the scalability of the current fabrication process and the need for standardization of manufacturing protocols.

A promising strategy to tackle both these topics could be represented by the use of advanced fabrication approaches, such as 3D printing [57, 58]. This technology could enable an accurate and repeatable fabrication process for the desired grafts with complex designs/geometries to better mimic the native structure and function of the acetabular labrum. Furthermore, this technology allows the development of advanced features, such as microporous internal architecture or functionalization of its surfaces that may improve graft integration with the surrounding host tissues.

The findings emerging from our study provide preclinical evidence supporting the use of a synthetic scaffold as an alternative to autograft or allograft tissue.

It is necessary to keep in mind that biomaterials are foreign bodies, thus adverse immune reactions to biomaterials might be a possible limitation. These adverse reactions commonly disrupt the healing process, resulting in immense pain for the patient, excessive inflammation, tissue destruction, and can lead to graft rejection [59, 60]. Therefore, evaluating the immune response to biomaterials in vitro may allow the determination of the likelihood of their acceptance in vivo.

Further studies are necessary to evaluate possible events of long‐term implant failure and the compatibility of these approaches with the required clinically approved materials, and to identify the most suitable fabrication for successful clinical translation of the prototypes toward clinical trials in human subjects. Additional tests should include assessment of the long‐term biocompatibility, host tissue integration, and tribological properties of the grafts.

## 5 Conclusion

Our study represents a pre‐clinical proof of concept to develop a synthetic graft for labral reconstruction with an “anatomical” profile to best restore the load bearing and ready to use in the surgical room for a more effective and rapid surgical technique. It might represent a new strategy to reduce the incidence of end‐stage HOA and the need for THR in adolescents and young adults.

## Author Contributions

**Enrico Tassinari:** conceptualization; methodology; investigation; graft prototypes design; arthroscopic procedure; writing – review and editing. **Mauro Petretta:** conceptualization; methodology; investigation; graft prototypes design and manufacturing; writing – review and editing; data analysis. **Giorgia Borciani:** in vitro experiments; writing – review and editing. **Luca Cristofolini:** graft prototypes design and manufacturing; mechanical test; writing – original draft; methodology; data analysis; writing – review and editing. **Eleonora Olivotto:** conceptualization; writing – original draft; methodology; graft prototypes design and manufacturing; writing – review and editing; data curation; project administration; supervision.

## Funding

This research was funded by the Italian Complementary National Plan PNC‐I.1 Research initiatives for innovative technologies and pathways in the health and welfare sector D.D. 931 of 06/06/2022, DARE ‐ DigitAl lifelong pRevEntion initiative, code PNC0000002, CUP: B53C22006450001.

## Disclosure

All authors listed meet the authorship criteria according to the latest guidelines of the International Committee of Medical Journal Editors, and they agree with the manuscript.

## Conflicts of Interest

The authors declare no conflicts of interest.

## Supporting information


**Data S1:** Supporting Information.

## Data Availability

The data that support the findings of this study are available from the corresponding author upon reasonable request.
