# Supplementary material for: Hip Labral Reconstruction With a Synthetic Graft: Development and Preclinical Validation
Source: Orthop Surg. 2026 Mar 20;18(4):791–800. doi: 10.1111/os.70246 (PMC13056495; doi:10.1111/os.70246)
Supplement: Supplementary file 1 — Data S1: Supporting Information. [file OS-18-791-s001.pdf]

Supplementary materials of the manuscript

Hip labral reconstruction with a synthetic graft: development and preclinical validation

By Enrico Tassinari, Mauro Petretta, Giorgia Borciani, Luca Cristofolini, Eleonora Olivotto

S1. Images of the reconstructions after completion of the mechanical tests

In the following pages, representative images of the labrum reconstructions after completion of the mechanical tests are reported. More details are reported in the Results section, under "Mechanical endurance of the graft". The images were acquired after test completion, focusing on possible damages (e.g. indents on the surface, scratches on the edges, and damage to the stitches). While some dark staining from the friction against the metal "femoral head" (especially in those specimens subjected to larger displacements), no damage was visible, in any of the specimens.

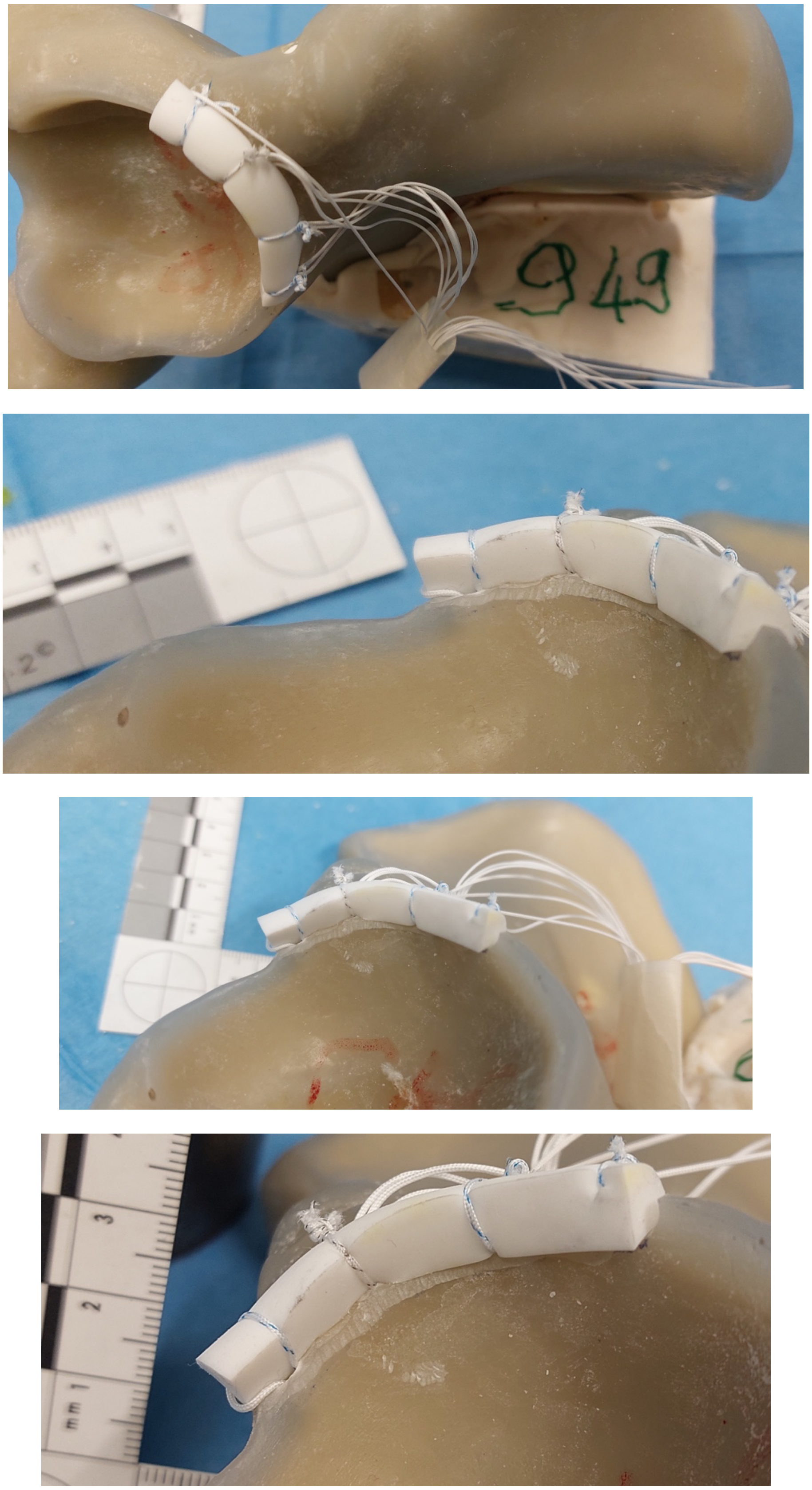

Fig. S1.1 *– Images from Specimen #949*
(after completing 10'000 cycles with 2.0 mm imposed displacement).

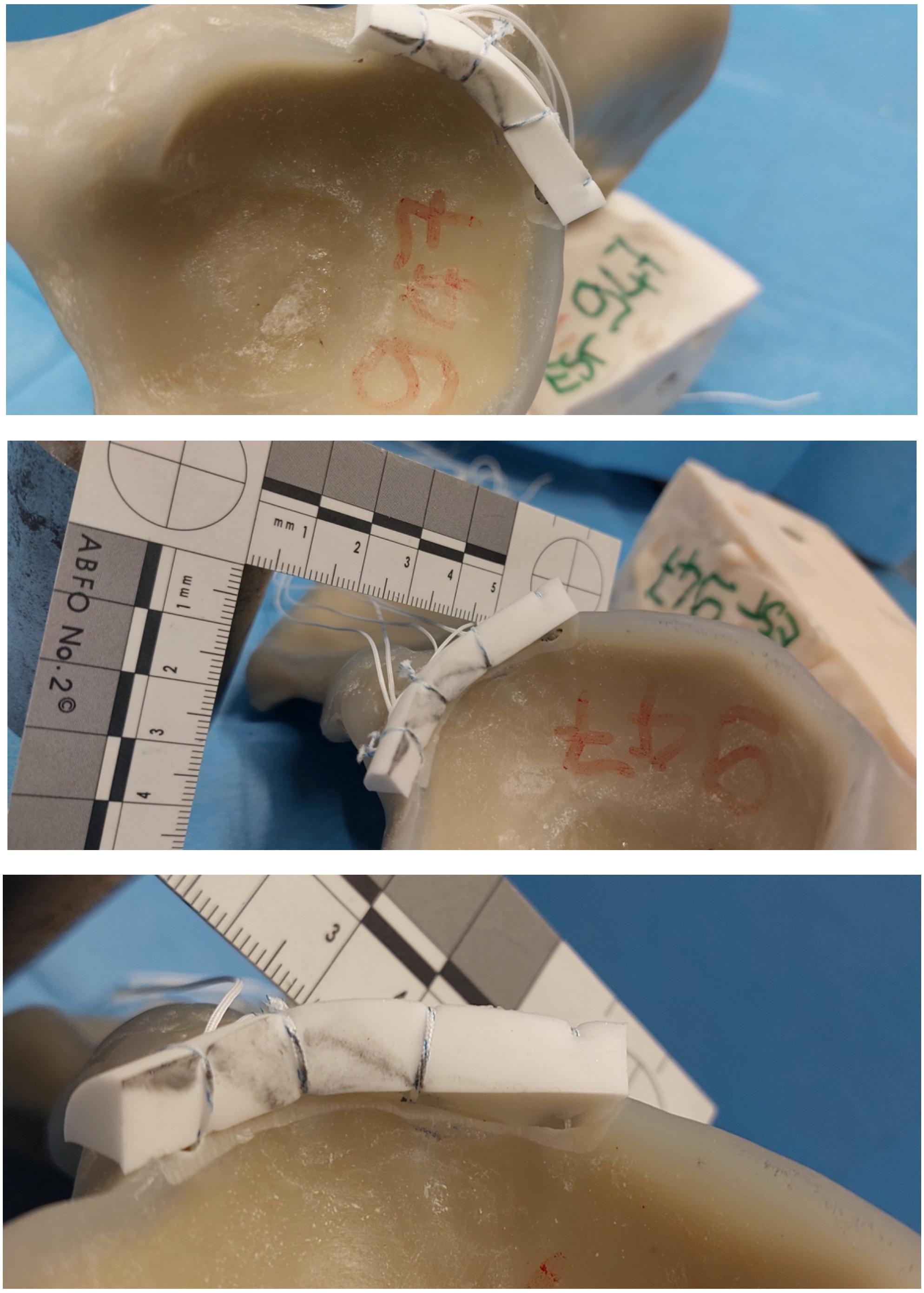

Fig. S1.2 *– Images from Specimen #947*
(after completing 10'000 cycles with 3.0 mm imposed displacement)

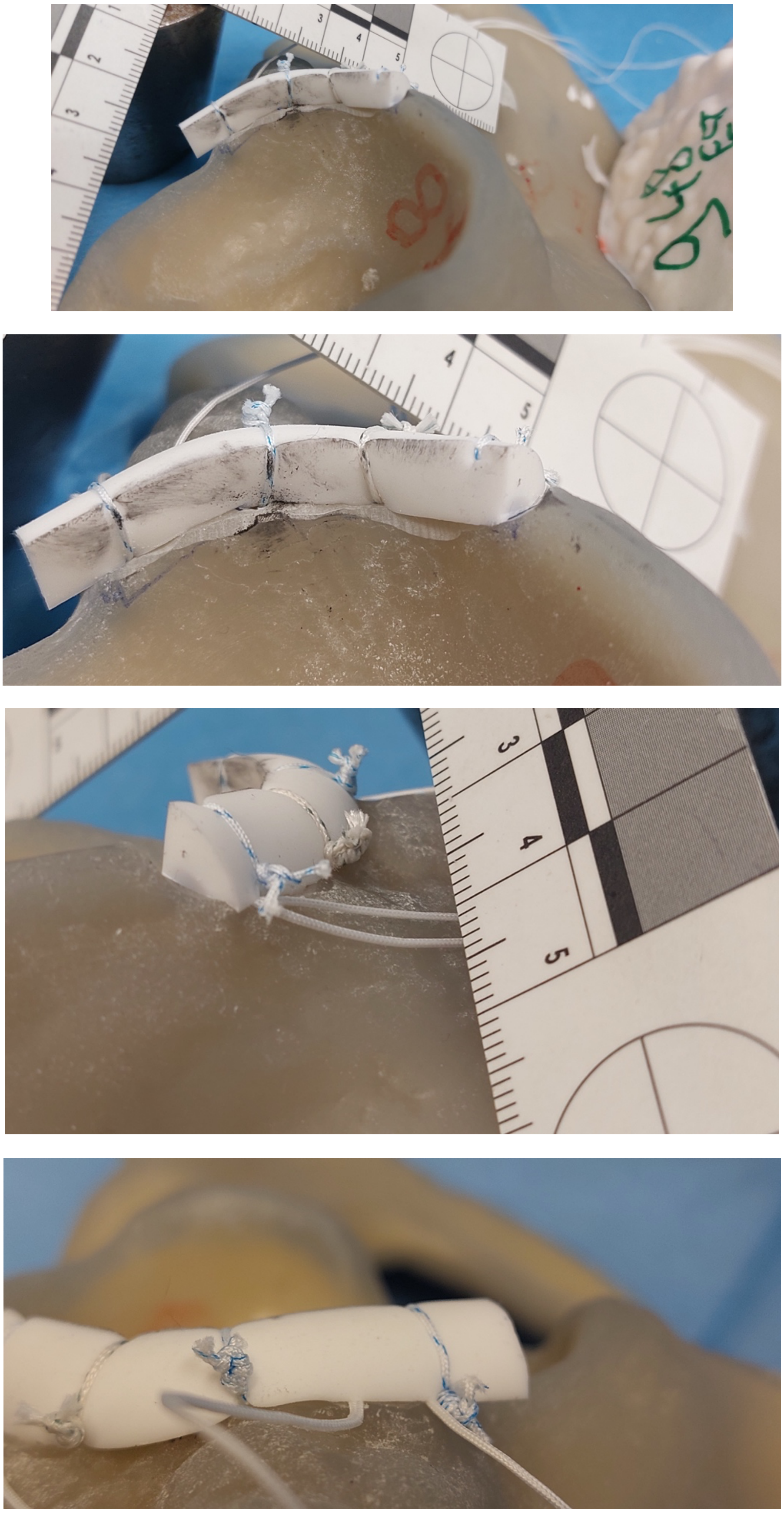

Fig. S1.3 *– Images from Specimen #948*
(after completing 10’000 cycles with 4.0 mm imposed displacement)

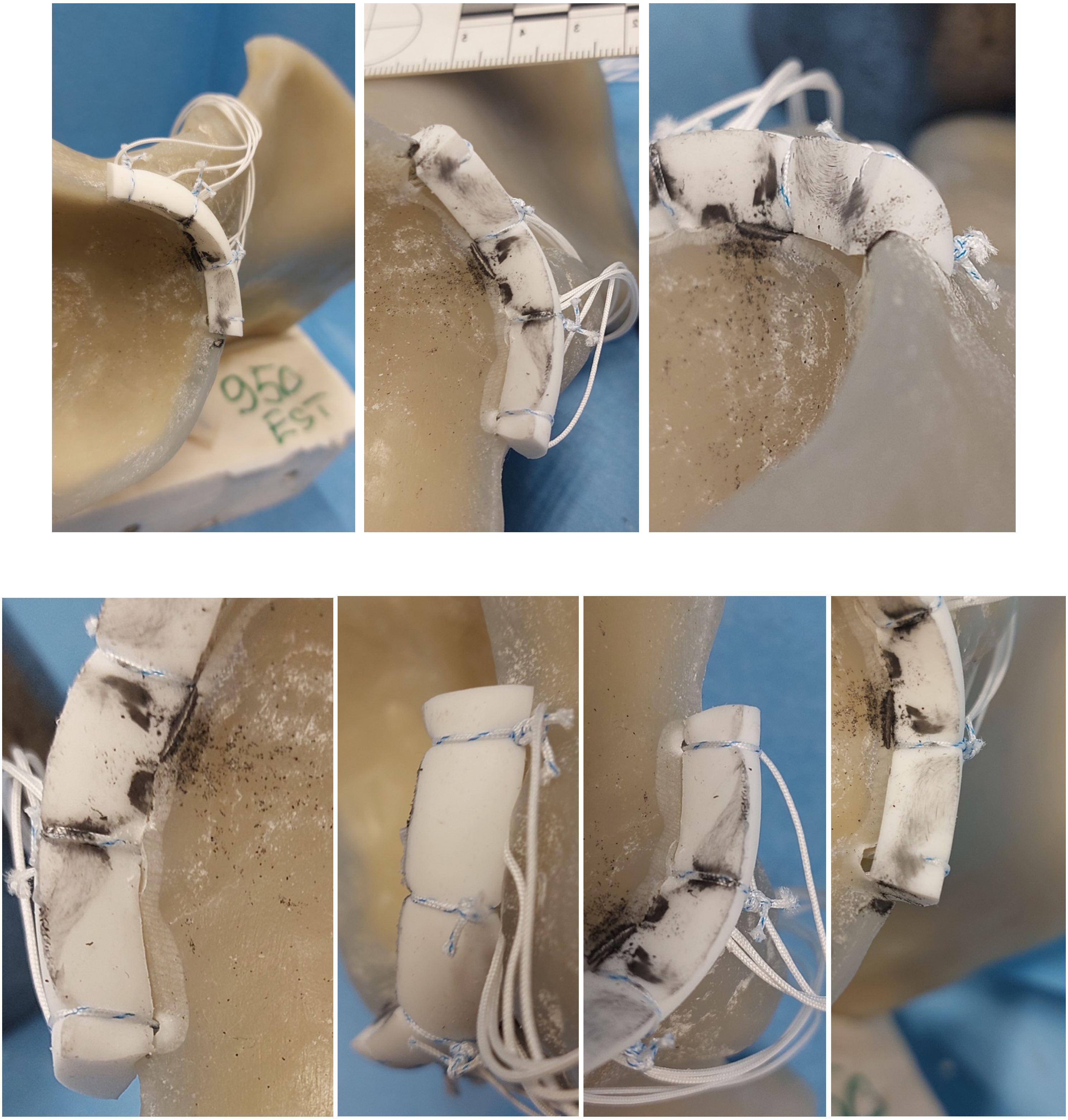

Fig. S1.4 *– Images from Specimen #950*
(after completing 10'000 cycles with 5.0 mm imposed displacement).
